# Supplementary material for: A survey on computational spectral reconstruction methods from RGB to hyperspectral imaging
Source: Sci Rep. 2022 Jul 13;12:11905. doi: 10.1038/s41598-022-16223-1 (PMC9279412; doi:10.1038/s41598-022-16223-1)
Supplement: Supplementary file 1 — Supplementary Information. [file 41598_2022_16223_MOESM1_ESM.docx]

Supplementary Information for

A survey on computational spectral reconstruction methods from RGB to hyperspectral imaging

**Jingang Zhang^1,+^ , Runmu Su^1,2,+^, Qiang Fu^5^, Wenqi Ren^4^, Felix Heide^5^ and Yunfeng Nie^6,*^**

^1^Intelligent Imaging Center & School of Future Technology, University of Chinese Academy of Sciences, Beijing, 100039;

^2^School of Computer Science and Technology, Xidian University, Xi’an, China, 710071; ^3^King Abdullah University of Science and Technology, Thuwal 23955-6900, Saudi Arabia;

^4^State Key Laboratory of Information Security, Institute of Information Engineering, Chinese Academy of Sciences, Beijing 100093;

^5^Computational Imaging Lab, Princeton University, NJ 08544, United States. ^3^Department of ^6^Brussel Photonics(B-PHOT), Applied Physics and Photonics, Vrije Universiteit Brussel, 1050 Brussels, Belgium;

+These authors contributed equally to this work

*Corresponding author email: [Yunfeng.Nie@vub.be](mailto:%20Yunfeng.Nie@vub.be)

This document provides the supplementary information to “A survey on computational spectral reconstruction methods from RGB to hyperspectral imaging”. The mathematical expressions of the loss functions mentioned in the original manuscript are given.

**Loss Function**
,

where$\mathcal{L}_{MSE}$ refers to mean square error, $H_{SR}$,$H_{GT}$ are the reconstructed and the ground truth hyperspectral image respectively.

,

where $\mathcal{L}_{MAE}$ is the mean absolute error.

,

where $\mathcal{L}_{eu}$ is the Euclidean error.

,

where $\mathcal{L}_{\triangle E}$ refers to the color error metric error.


,

where $\mathcal{L}_{mper}$ represents slightly modified perceptual loss function, $\phi_{j}$ is the feature maps of the j-th layer of the VGG16 network, $C_{j}$, $H_{j}$ and $W_{j}$ indicates the channel, height and width of the feature maps, G is Gram matrix.

,

where $\mathcal{L}_{GAN}$ represents the Conditional Generative Adversarial loss, G, D denote generator and discriminator respectively, $I_{RGB}$ is RGB image.

,

where $\mathcal{L}_{adv}$ refers to the Wasserstein GAN loss.

,

where$\left\| \cdot\right\|_{1}$ is the L1 norm.

,

where $\left\| \cdot\right\|_{2}$ is the L2 norm.

,

where $\mathcal{L}_{MRAE}$ is mean the relative absolute error.

,

where $\mathcal{L}_{CSS}$ refers to the camera spectral sensitivity (CSS) loss, $\Phi$ is the CSS function.

,

where, $\mathcal{L}_{SSIM}$ is the structural similarity metric, $\mu_{r}$ and $\mu_{g}$ are the mean values of the reconstructed and ground truth hyperspectral images respectively, $\sigma_{r}$ and $\sigma_{g}$ are the variances, and $\sigma_{r,g}$ is the covariance of the reconstructed and the ground truth hyperspectral images.
